# Decision Tree Modeling to Predict Myopia Progression in Children Treated with Atropine: Toward Precision Ophthalmology

**DOI:** 10.3390/diagnostics15162096

**Published:** 2025-08-20

**Authors:** Jun-Wei Chen, Chi-Jie Lu, Chieh-Han Yu, Tzu-Chi Liu, Tzu-En Wu

**Affiliations:** 1 Department of Medical Education, Linkou Chang Gung Memorial Hospital, Guishan Dist., Taoyuan City 333, Taiwan; alexjunwei1102@gmail.com; 2 Graduate Institute of Business Administration, Fu Jen Catholic University, New Taipei City 242, Taiwan; 059099@mail.fju.edu.tw (C.-J.L.);; 3 Artificial Intelligence Development Center, Fu Jen Catholic University, New Taipei City 242, Taiwan; 4 Department of Information Management, Fu Jen Catholic University, New Taipei City 242, Taiwan; 5 School of Medicine, Chang Gung University, Taoyuan City 333, Taiwan; 6 Department of Ophthalmology, Shin Kong Wu Ho-Su Memorial Hospital, Taipei 111, Taiwan; 7 School of Medicine, Fu Jen Catholic University, New Taipei City 242, Taiwan

**Keywords:** myopia, atropine, machine learning, decision tree, precision ophthalmology

## Abstract

**Background/Objectives**: Myopia is a growing global health concern, especially among school-aged children in East Asia. Topical atropine is a key treatment for pediatric myopia control, but individual responses vary, with some children showing rapid progression despite higher doses. This retrospective observational study aims to develop an interpretable machine learning model to predict individualized treatment responses and support personalized clinical decisions, based on data collected over a 3-year period without a control group. **Methods**: A total of 1545 pediatric eyes treated with topical atropine for myopia control at a single tertiary medical center are analyzed. Classification and regression tree (CART) is constructed to predict changes in spherical equivalent (SE) and identify influencing risk factors. These factors are mainly received treatments for myopia including atropine dosage records, treatment duration, and ophthalmic examinations. Furthermore, decision rules that closely resemble the clinical diagnosis process are provided to assist clinicians with more interpretable insights into personalized treatment decisions. The performance of CART is evaluated by comparing with the benchmark model of least absolute shrinkage and selection operator regression (Lasso) to confirm the practicality of CART usage. **Results**: Both the CART and Lasso models demonstrated comparable predictive performance. The CART model identified baseline SE as the primary determinant of myopia progression. Children with a baseline SE more negative than −3.125 D exhibited greater myopic progression, particularly those with prolonged treatment duration and higher cumulative atropine dosage. **Conclusions**: Baseline SE has been identified as the key factor affecting SE difference. The generated decision rules from CART demonstrate the use of explainable machine learning in precision myopia management.

## 1. Introduction

Myopia has emerged as a global public health priority due to its rising prevalence, particularly among school-aged children in East Asia [1,2]. Early-onset and rapidly progressing myopia significantly increase the risk of irreversible vision-threatening complications such as myopic maculopathy, glaucoma, and retinal detachment [3]. The growing burden of high myopia underscores the need for timely detection, risk stratification, and evidence-based interventions to prevent long-term visual impairment [4,5].

Pharmacological management, particularly the use of topical atropine, has gained widespread recognition as a cornerstone of pediatric myopia control [6]. Numerous randomized controlled trials and meta-analyses have demonstrated that concentrations between 0.01% and 0.05% are effective in slowing myopic progression while minimizing side effects such as photophobia and accommodation loss [7,8]. Despite its proven efficacy, interindividual variability in treatment response remains a significant challenge, with some children continuing to experience rapid progression despite adherence to more aggressive therapy [9,10,11].

This variability highlights the urgent need to identify reliable predictors of treatment response [12,13,14]. Previous studies have pointed to several clinical and biometric factors such as baseline spherical equivalent (SE), axial length, age, cumulative dosage, and treatment duration as key determinants of therapeutic efficacy [15,16]. For example, Du et al. found that children with lower baseline myopia and older age showed better outcomes with weekly 1% atropine [15], while Rose et al. demonstrated that axial length at baseline is a critical predictor of treatment effect [16].

Recent guidelines from the International Myopia Institute (IMI) and European Society of Ophthalmology have advocated for data-driven and algorithm-supported approaches to myopia management [17,18,19,20], but few studies have incorporated explainable artificial intelligence (AI) models into treatment planning. The current literature has rarely addressed how real-world variables including baseline SE, treatment dosage, duration, and intraocular pressure interact to influence refractive outcomes in atropine-treated children [21,22,23].

The integration of AI and machine learning (ML) in ophthalmology offers novel opportunities for advancing precision medicine [24,25]. ML methods offer distinct advantages over traditional statistical models, as they take nonparametric, data-driven approaches and are capable of capturing complex variable interactions more effectively [24]. Furthermore, decision tree-based ML methods, such as classification and regression tree (CART), provide interpretable decision rules that offer useful insights closely resembling the clinical diagnosis process. This can assist clinicians by presenting clear, evidence-based decision paths and potential outcomes, thereby supporting more informed treatment decisions [24,25,26].

In this context, the CART model offers a more intuitive framework for clinicians, facilitating personalized treatment adjustments based on individual patient profiles. Therefore, our study implements the CART model and utilizes a large real-world dataset to identify factors influencing refractive change in children undergoing atropine therapy. Key variables related to myopia treatment including atropine dosage records, treatment duration, and ophthalmic examinations are incorporated into the CART model to establish a clinically interpretable and personalized approach that provides both decision rules and predictions for SE differences. By prioritizing interpretability without compromising predictive power, our study addresses a critical gap in pediatric myopia research and highlights the potential of decision tree models to support individualized treatment strategies. As the global prevalence of myopia continues to rise, especially in younger populations, AI-assisted clinical tools such as ours will become increasingly essential in advancing the paradigm of precision myopia care.

## 2. Methodology

### 2.1. Study Design and Population

**Figure 1:**
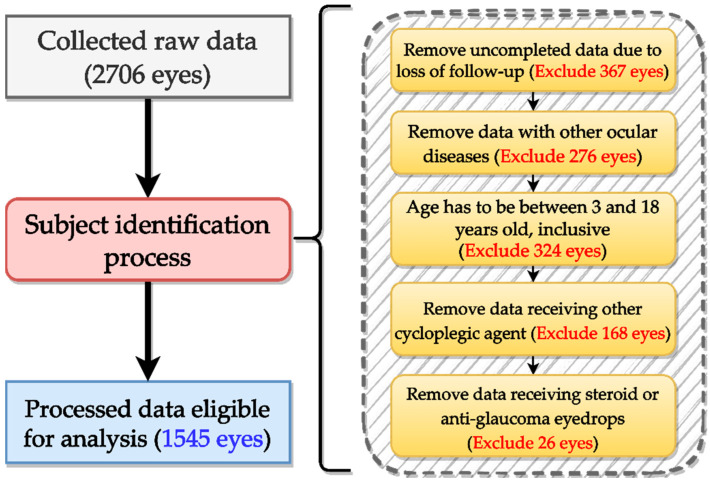
Subject identification flow chart.

This retrospective observational study analyzed de-identified clinical data collected at the Department of Ophthalmology, Shin Kong Wu Ho-Su Memorial Hospital (Taipei, Taiwan). The dataset was obtained from pediatric patients who received topical atropine treatment for myopia control between 1 January 2005 and 31 December 2008. A total of 2706 eyes were initially reviewed, then a subject identification process is performed as visualized in Figure 1. The process aims to remove subjects that are irrelevant to this study and includes the following criteria: (1) incomplete data due to loss to follow-up (exclude 367), (2) presence of other ocular diseases (exclude 276), (3) age outside the 3–18-year range (exclude 324), (4) concurrent use of other cycloplegic agents (exclude 168), and (5) prescription of corticosteroids or anti-glaucoma medications (exclude 26). After the subject identification process, a total of 1545 eyes were deemed eligible for analysis. The study protocol was reviewed and approved by the Institutional Review Board of Shin Kong Wu Ho-Su Memorial Hospital (IRB No. 20220706R).

### 2.2. Clinical Measurements

Non-contact tonometry (Xpert NCT plus, Reichert, Leica Inc., Midview City, Singapore) was performed with patients in a sitting position, without local anesthesia. Refractive errors were measured using an autorefractometer keratometer (Canon RK5, Canon Inc., Tochigiken, Japan). The target variable was the change in myopic spherical equivalent (SE difference), calculated by subtracting the baseline SE from the SE measured at the final follow-up visit. Additional predictor variables included baseline IOP, IOP difference, gender, age at baseline, total treatment duration, total cumulative dosage of atropine, and average monthly atropine dosage. Although SE was the primary outcome, intraocular pressure (IOP) was also incorporated as a predictive variable and further analyzed in the model interpretation. The total treatment duration was defined as the time interval between the first atropine prescription and the final clinic visit, within the study window of 1 January 2005 to 31 December 2008. At each visit, ophthalmologists prescribed atropine eye drops at concentrations of 1.0%, 0.5%, 0.25%, or 0.1% (Oasis Chemical Ind. Co., Thane, Maharashtra, India), based on clinical judgment of myopia severity. Corresponding dosages per bottle were 50 mg, 25 mg, 12.5 mg, and 5 mg, respectively. The dosage prescribed at each visit was calculated as the product of the number of bottles and the atropine content per bottle. The total cumulative dosage was computed by summing all prescribed atropine amounts across visits. The average monthly dosage was derived by dividing the total cumulative dosage by the number of treatment months, providing a normalized monthly exposure measure. The demographic of target and predictor variables are presented in Table 1.

### 2.3. Machine Learning Methods

To provide insights into how factors affecting SE difference, classification, and regression tree (CART) are utilized in this study. CART is a decision tree-based method that is capable of generating decision rules with a tree structure [27]. For regression task, CART builds a tree by recursively splitting the dataset into subsets that minimize prediction error, building a binary decision tree structure in the process. At each node, CART evaluates all possible splits across the available features and identifies the split thresholds that results in the lowest prediction error. The splitting threshold is determined through the sum of squared error (SSE) as shown in Equation (1):

(1) $$SSE=\sum_{i\in S_{1}}{\left(y_{i}-{\bar{y}}_{S_{1}}\right)}^{2}+\sum_{i\in S_{2}}{\left(y_{i}-{\bar{y}}_{S_{2}}\right)}^{2}$$

where $S_{1}$ and $S_{2}$ denotes the potential binary splitting subsets of data, whereas ${\bar{y}}_{S_{1}}$ and ${\bar{y}}_{S_{2}}$ are the mean of the corresponding subsets. SSE measures the differences between the actual target values and their mean within a node. By selecting the split that minimizes SSE, the algorithm ensures that each decision rule contains observations with more similar outcomes that are close to actual target values. Once the tree is fully constructed, predictions for new instances are made by passing them through the decision rules of the tree until they reach a leaf node, where the average of the target values within that node is used as the final predicted outcome. This enables CART to capture complex, non-linear relationships in regression data while maintaining interpretability through its decision tree structure.

Least absolute shrinkage and selection operator regression (Lasso) is used as benchmark method to evaluate CART performance as it is commonly used in many ophthalmology studies. Lasso extends the basic regression by employing a penalty term that shrinks the coefficient of less influential feature on target outcome to zero [28]. This approach selects the important feature and maintains the effectiveness of the model. Assuming that a total of P predictor features are used to construct a Lasso model, the loss function of Lasso is denoted in the following Equation (2):

(2) $$argmin\left\{\sum_{i=1}^{n}(Y_{i}-\sum_{j=1}^{P}\beta_{j}x_{i,j}{)}^{2}+\lambda \sum_{j=1}^{P}\left|\beta_{j}\right|\right\}$$

where λ is a parameter denoting the regularization strength of penalty and $\beta_{j}$ is the coefficient of the *j*th predictor feature.

### 2.4. Modeling Scheme

**Figure 2:**
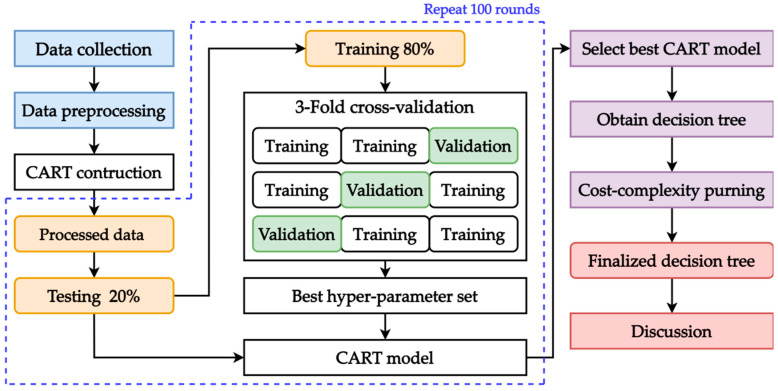
Model construction scheme.

The scheme for constructing the CART model is presented in Figure 2. The collected data, after completing the subject identification process, is then preprocessed to ensure the quality of the dataset. Data preprocessing includes both abnormal value verification and extreme value inspection, with no such issues identified in the data. Once the data is cleaned, it is divided into training and testing sets for constructing the CART model. During model construction, 3-Fold cross validation (3FCV) is deployed to further divide the training set into training and validation sets for hyper-parameter tuning. 3FCV randomly divides the training set into three equal-sized portions, then the model is built from three of the two portions and validated with the remaining one until all portions are validated once. The candidate hyper-parameter sets are evaluated during this phase, which the optimal set is the one with the best validation performance. Next, the model will be rebuilt with the full-length training set and the corresponding optimal hyper-parameter set for testing. This model construction process repeats 100 rounds to ensure the robustness and stability of the model results.

After completing construction of the CART models, the testing results are evaluated to select the model with the best performance. Once the best model is identified, an initial decision tree is generated from it. Since the goal of this study is to provide deeper insights into the factors affecting SE differences to support clinical diagnosis, the initial tree is further simplified through cost-complexity pruning to obtain a more interpretable tree that is easier for clinicians to understand. Cost-complexity pruning simplifies a decision tree by removing branches that contribute little to prediction, aiming to balance model simplicity and performance. Thus, the finalized decision tree can be obtained and visualized as a tree plot for interpreting decision rules.

In addition, Lasso is built following the same model construction process and acts as the benchmark method for performance comparison with the CART model. Five common error metrics are used in this study for performance evaluation, namely mean squared error (MSE), root mean squared error (RMSE), mean absolute error (MAE), relative absolute error (RAE), and relative squared error (RSE).

## 3. Results

In this study, both Lasso and CART were employed to predict changes in SE in children undergoing atropine treatment for myopia control. The predictive performance of the two models are close, as presented in Table 2. The results are displayed in mean and standard deviation. Lasso demonstrates a slightly better MSE of 0.287, RMSE of 0.535, and MAE of 0.392. In contrast, CART yields an MSE of 0.296, RMSE of 0.543, and MAE of 0.398. Similarly, the RAE and RSE values are slightly better in the Lasso model (RAE: 0.917, RSE: 0.853) compared to those of CART (RAE: 0.931, RSE: 0.882). These results indicate that both models offer adequate predictive accuracy, with only slight numerical differences.

**Figure 3:**
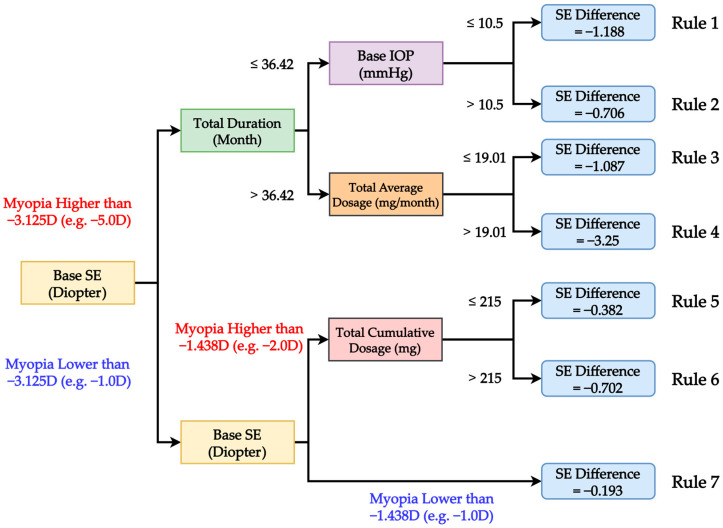
Tree plot illustrates CART model decision rules based on baseline SE, treatment duration, dosage, and IOP.

While both models have similar performance, with Lasso slightly outperforming CART, the results confirm that CART can produce reasonable prediction results. The goal of this study is to provide more useful insights supporting clinical diagnosis; CART is the utilized model as it is capable of providing tree-like decision rules. The structure of the CART model enables clinicians to visualize decision paths. This rule-based representation not only simplifies complex relationships among variables but also enhances clinical decision-making by offering a transparent rationale for predicting treatment outcomes. In real-world clinical settings, where explainability is critical for gaining trust and guiding personalized care, this interpretability becomes a major strength. Figure 3 visualized the best decision tree from CART. As shown in the figure, a decision rule composes of a path from the root node (the first node from the left of the tree) to the leaf node (the blue nodes located at the right of the tree), while the conditions within the path will decide which leaf node a subject belongs to. Based on the decision tree, baseline myopic SE was the primary splitting variable. For clinical interpretation, we followed three established principles: (1) more negative SE indicates more severe myopia, (2) more negative SE difference denotes poorer treatment outcome, and (3) although myopic SE values are mathematically negative, clinical severity is interpreted based on their absolute magnitude. The decision tree model initially stratified patients into two major subgroups using a threshold of −3.125 D. Patients with myopic SE higher than −3.125 D (e.g., −5.00 D) were categorized as having more severe baseline myopia, while those with myopic SE lower than −3.125 D (e.g., −1.00 D) were considered less myopic. In the latter subgroup, a secondary split at −1.438 D further delineated moderate and mild myopia. The decision rules in Figure 2 are further organized into Table 3 for easy interpretation.

In patients with more severe baseline myopia (myopic SE higher than −3.125 D, e.g., −5.0 D), four decision rules (Rules 1–4) were identified. For those with shorter treatment duration (≤36.42 months) and lower intraocular pressure (IOP ≤ 10.5 mmHg), the predicted SE difference was −1.188 D (Rule 1), indicating significant myopic progression. When baseline IOP > 10.5 mmHg under the same treatment duration, progression was slightly less severe, with a predicted SE difference of −0.706 D (Rule 2). Among those with prolonged treatment duration (>36.42 months) and lower average monthly dosage (≤19.01 mg/month), the SE difference was −1.087 D (Rule 3). Notably, in patients who had both longer treatment duration and higher average dosage (>19.01 mg/month), the model predicted the most pronounced myopic worsening, with an SE difference of −3.25 D (Rule 4), suggesting that even intensive treatment may not sufficiently control progression in high-risk cases. In the subgroup with less severe baseline myopia (myopic SE lower than −3.125 D, e.g., −1.0 D or −2.0 D), three additional decision rules (Rules 5–7) were identified. Patients with moderate myopia (myopic SE between −3.125 D and −1.438 D, e.g., −2.0 D) and lower cumulative dosage (≤215 mg) had a predicted SE difference of −0.382 D (Rule 5), whereas those receiving higher cumulative dosage (>215 mg) had a more negative SE difference of −0.702 D (Rule 6), indicating that despite more aggressive treatment, progression still occurred in this group. In contrast, patients with mildest myopia (myopic SE lower than −1.438 D, e.g., −1.00 D) had the most favorable outcome, with a minimal predicted SE difference of −0.193 D (Rule 7).

These results collectively underscore the importance of baseline myopia severity in determining treatment response. While longer duration and higher dosage may mitigate atropine control in high myopic subgroups, patients with more severe baseline myopia remain at higher risk of deterioration, even under intensified treatment regimens. The CART model provides interpretable decision rules that can support individualized treatment planning based on baseline refractive status, treatment intensity, and ocular characteristics.

## 4. Discussion

Myopia represents a significant global public health concern, particularly due to its rising prevalence in children and the associated risk of vision-threatening ocular complications. Developing effective strategies to curb pediatric myopia progression is therefore essential. Among pharmacological interventions, topical atropine has emerged as a leading treatment; however, considerable variability in individual treatment responses highlights the need to identify predictive factors that can optimize therapeutic outcomes. Consistent with previous findings indicating that severe baseline myopia is associated with a higher risk of treatment failure or accelerated progression [8,15], the present study employed real-world data and the CART algorithm to identify clinical variables influencing changes in SE in children undergoing atropine therapy for myopia control.

This study employed a CART model to identify and visualize key predictors of myopic progression in children receiving topical atropine treatment. Among the evaluated variables, baseline SE emerged as the most influential determinant of treatment response, a finding that aligns with the recent literature on atropine efficacy and myopia risk stratification [1,4]. By stratifying patients based on a critical SE threshold of −3.125 D, the model offered a clinically interpretable framework for understanding how initial refractive status influences myopia control outcomes.

Notably, patients with baseline myopic SE higher than −3.125 D (e.g., −5 D) demonstrated significantly greater myopic progression, with predicted SE changes ranging from −0.706 D to −3.25 D, modulated by treatment duration, cumulative dosage, and IOP. In contrast, those with baseline SE equal to or less than −3.125 D (e.g., −1 D) showed more favorable treatment responses, with predicted SE differences ranging from −0.382 D to −0.193 D. These findings are consistent with the report by Jonas et al., which emphasized the association between higher baseline myopia and faster progression [18], as well as the analysis by Chen et al. (2024), where SHAP values similarly underscored baseline SE as a dominant predictor of atropine treatment efficacy [21].

Among all subgroups identified by the CART model, Rule 7 exhibited the most effective control of myopic progression. This subgroup comprised children with a baseline SE lower than −3.125 D and further stratified to those with SE below −1.438 D. The mean SE difference in this group was merely −0.193 D, representing excellent control. Notably, Rule 7 did not explicitly stratify by atropine dosage, yet patients in this category achieved favorable outcomes. This suggests that children with milder baseline myopia may attain stable refractive outcomes even under less intensive treatment protocols.

These findings highlight the potential of early intervention in low-risk patients and are consistent with the results of Rose et al., who reported that reduced axial elongation (<0.2 mm/year) under low-dose atropine was associated with meaningful clinical benefit [16]. In particular, patients with myopic SE below −1.438 D showed minimal progression despite conservative dosing strategies. This supports the notion that individuals with less severe refractive errors may not require high-intensity atropine regimens to achieve effective myopia control [6].

Another subgroup demonstrating favorable myopia control was Rule 5, which included patients with baseline SE between −3.125 D and −1.438 D and a cumulative atropine dosage of ≤215 mg. The SE progression of −0.382 D in this group was slightly higher than that observed in Rule 7, yet still within the range considered clinically effective for myopia control. Together, these two subgroups support the principle that children with moderate baseline myopia can achieve satisfactory refractive stability through appropriately titrated atropine dosing.

In contrast to the well-controlled subgroups, Rule 6 identified treatment groups with similar baseline SE (between −3.125 D and −1.438 D) but with a cumulative atropine dosage exceeding 215 mg. These patients experienced worse control with an SE progression of −0.702 D. This disparity suggests the possibility of overtreatment or confounding factors such as poor adherence, individual pharmacodynamic variability, or rebound effects. These internal comparisons align with the dose–response plateau phenomenon described in recent meta-analyses by Wang et al. and Hou et al., which caution that increasing atropine doses does not always correspond to proportional therapeutic gains [7,8].

Importantly, the decision pathways outlined in Rules 3 and 4 underscore the interplay between average dosage and treatment duration. While neither variable alone reliably predicts outcomes, their interaction plays a pivotal role in shaping refractive change. This highlights the importance of consistent therapeutic exposure and adherence to treatment regimens. These findings reinforce the rationale behind existing treatment algorithms that emphasize individualized dosage titration and ongoing monitoring an approach supported by recent expert consensus statements from the European Society of Ophthalmology [20,29].

At the more severe end of the spectrum, Rule 4 included patients with baseline SE greater than −3.125 D, extended treatment durations (>36.42 months), and high average monthly atropine dosages (>19.01 mg/month). This subgroup exhibited the most pronounced myopic progression, with an average SE change of −3.25 D. These results clearly indicate that more aggressive treatment in high-risk patients does not necessarily translate into better control and may even be associated with continued deterioration. This finding echoes concerns raised by Du et al., who warned against assuming linear benefits with prolonged atropine therapy in children with high baseline myopia [15]. Moreover, our findings resonate with Hou et al.’s meta-analysis, which reported a plateau or even a decline in efficacy beyond certain atropine dosage thresholds [7].

Together, these observations emphasize the complexity of managing high-risk myopia and underscore the need for individualized treatment approaches that consider not only baseline refractive error but also treatment duration, dosage, and patient adherence.

In addition to refractive error and treatment intensity, baseline intraocular pressure (IOP) and patient compliance emerged as key factors influencing treatment outcomes. Rules 1 and 2, which involved patients with baseline SE greater than −3.125 D and shorter treatment durations (≤36.42 months), revealed that baseline IOP played a moderating role. Specifically, patients with lower baseline IOP (≤10.5 mmHg) exhibited greater myopic progression (SE change = −1.188 D) compared to those with IOP >10.5 mmHg (SE change = −0.706 D). This suggests that reduced IOP may be associated with suboptimal treatment response in certain subgroups. The CART model effectively captured these nuanced interactions between treatment duration, IOP, and dosage, underscoring the importance of pre-treatment IOP assessment. Moreover, prior studies have highlighted the potential influence of IOP and ocular biomechanical properties such as scleral rigidity and axial elongation dynamics on atropine responsiveness. The lower Base IOP may reflect diminished ocular rigidity, particularly in pediatric eyes. This reduced mechanical resistance could facilitate excessive scleral expansion under environmental and visual demands, leading to axial elongation and worsening myopia [30,31]. Younger patients often have lower IOP and simultaneously possess more pliable scleral tissue, which might render atropine’s anti-elongation effects less effective. This interaction underscores the need for early intervention strategies tailored to ocular biomechanical profiles [32].

Demographic factors such as age and gender had minimal influences on SE outcomes, consistent with prior findings from Rose et al. and Li et al. [16,33]. Jonas et al. likewise emphasized that therapeutic responses are more driven by optical and pharmacologic parameters than by age or sex [18].

Clinically, our findings advocate for the implementation of individualized myopia control strategies. For children with baseline myopic SE higher than −3.125 D, early identification combined with a carefully calibrated balance of efficacy and safety in atropine dosing is essential. Conversely, patients with mild-to-moderate myopia appear to benefit most from prompt yet conservative treatment approaches characterized by shorter durations and lower dosages without compromising outcomes. These insights are in line with recent modeling studies that support stratified treatment regimens based on initial refractive severity and predicted responsiveness [21,22].

Moreover, our results complement the growing body of research aimed at personalizing myopia management through baseline biometric profiling. Emerging studies incorporating axial length, choroidal thickness, and artificial intelligence-based models have demonstrated promising potential in forecasting treatment efficacy [11,33,34]. The SE-based stratification framework presented in this study could be integrated with these parameters to support more robust, multi-parametric prediction models, ultimately enhancing individualized therapeutic decision-making.

Several limitations of this study should be acknowledged. First, the retrospective single-center design may introduce selection bias and limit the generalizability of findings to broader populations. Although the dataset was clinically rich, it originated from a single institutional source, and external validation with independent cohorts is needed to confirm the robustness of the model. Second, while the CART model offers intuitive interpretability, it may be susceptible to overfitting or sensitivity to small variations in the dataset. Future studies incorporating ensemble methods or hybrid machine learning frameworks could enhance stability and predictive performance [3].

Moreover, the absence of key biometric and behavioral variables such as axial length, choroidal thickness, screen time, and outdoor exposure limits the comprehensiveness of the predictive framework. In particular, axial length is increasingly regarded as a gold standard for assessing structural changes in myopia progression, and its inclusion will be a priority in future model iterations [16]. Lastly, the use of SE difference at a single time point as the primary outcome restricts the ability to evaluate longitudinal treatment trajectories, emphasizing the need for dynamic modeling in future research. Additionally, we explored the possibility of using ratio metrics such as SE difference divided by baseline SE. However, due to the negative values of SE and resulting interpretability challenges, these ratios were not adopted in the final model.

Our study affirms the utility of decision tree models in parsing complex clinical data to guide myopia control strategies. The CART model identified baseline SE (−3.125 D) as the most critical predictor of treatment response, with clear dosage and IOP interaction pathways further refining outcome prediction. These findings support the development of individualized, dosage-sensitive atropine treatment plans. With future integration of biometric and genetic markers, such rule-based models may play a pivotal role in advancing precision myopia medicine.

## 5. Conclusions

This study demonstrates the clinical utility of an explainable artificial intelligence CART model in predicting individualized refractive outcomes among children undergoing atropine treatment for myopia control. By stratifying patients based on baseline SE, treatment duration, dosage, and IOP, the CART model identified seven interpretable decision rules with clear clinical implications. Baseline SE emerged as the primary predictor, with children presenting with SE higher than −3.125 D showing greater risk of myopic progression despite intensive therapy. Therefore, early intervention among children with less severe baseline myopia is advisable, as they tend to show better responses even under less intensive treatment. In contrast, those with milder baseline myopia achieved favorable refractive stability even with lower treatment intensity. The decision tree offers a transparent, visual framework that facilitates personalized treatment planning and enhances physician trust. This study highlights the potential of interpretable machine learning to bridge data-driven analytics with clinical decision-making, setting the foundation for precision myopia management through AI-assisted tools.

## Figures and Tables

**Table 1:** Variable demographic.

Variable	Mean (SD)
Target Variable	
SE Difference	−0.46 (0.58)
Numerical Variable	
Base SE (Diopter)	−2.48 (1.57)
Base IOP (mmHg)	14.51 (2.69)
IOP Difference (mmHg)	0.57 (2.48)
Age (y/o)	10.53 (2.56)
Total Duration (Month)	20.02 (12.01)
Total Cumulative Dosage (mg)	118.72 (133.74)
Total Average Dosage (mg/month)	6.43 (6.27)
Categorical Variable	
Gender	N (%)
Male	813 (53%)
Female	732 (47%)

**Table 2:** 100-round average results of Lasso and CART.

Model	MSE	RMSE	MAE	RAE	RSE
Lasso	0.287 (0.033)	0.535 (0.031)	0.392 (0.016)	0.917 (0.030)	0.853 (0.044)
CART	0.296 (0.034)	0.543 (0.031)	0.398 (0.017)	0.931 (0.036)	0.882 (0.068)

**Table 3:** Decision rules extracted from the CART model, specifying combinations of clinical features and corresponding predicted average SE differences.

Rules	Combination of Condition	SE Prediction
1	Base SE Higher than −3.125 D and Total Duration ≤ 36.42 months and Base IOP ≤ 10.5 mmHg	−1.188
2	Base SE Higher than −3.125 D and Total Duration ≤ 36.42 months and Base IOP > 10.5 mmHg	−0.706
3	Base SE Higher than −3.125 D and Total Duration > 36.42 months and Total Average Dosage ≤ 19.01 mg/month	−1.087
4	Base SE Higher than −3.125 D and Total Duration > 36.42 months and Total Average Dosage > 19.01 mg/month	−3.25
5	Base SE Lower than −3.125 D and Base SE Higher than −1.438 D and Total Cumulative Dosage ≤ 215 mg	−0.382
6	Base SE Lower than −3.125 D and Base SE Higher than − 1.438 D and Total Cumulative Dosage > 215 mg	−0.702
7	Base SE Lower than −3.125 D and Base SE Lower than −1.438 D	−0.193

## Data Availability

The datasets generated during and/or analyzed during the current study are not publicly available due to privacy/ethical restrictions but are available from the corresponding author on reasonable request.

## References

[1] Zhang X.-J., Zaabaar E., French A.N., Tang F.-Y., Kam K.-W., Tham C.C., Chen L.-J., Pang C.-P., Yam J.C. (2025). Advances in myopia control strategies for children. Br. J. Ophthalmol..

[2] Zhang X., Zhou Y., Wang Y., Du W., Yang J. (2023). Trend of myopia through different interventions from 2010 to 2050: Findings from Eastern Chinese student surveillance study. Front. Med..

[3] Bullimore M.A., Ritchey E.R., Shah S., Leveziel N., Bourne R.R.A., Flitcroft D.I. (2021). The risks and benefits of myopia control. Ophthalmology.

[4] Eppenberger L.S., Grzybowski A., Schmetterer L., Ang M. (2024). Myopia Control: Are We Ready for an Evidence Based Approach?. Ophthalmol. Ther..

[5] Fricke T.R., Sankaridurg P., Naduvilath T., Resnikoff S., Tahhan N., He M., Frick K.D. (2023). Establishing a method to estimate the effect of antimyopia management options on lifetime cost of myopia. Br. J. Ophthalmol..

[6] Wei X.-L., Wu T., Dang K.-R., Hu K.-K., Lu X.-T., Gong M., Du Y.-R., Hui Y.-N., Tian X.-M., Du H.-J. (2023). Efficacy and safety of atropine at different concentrations in prevention of myopia progression in Asian children: A systematic review and Meta-analysis of randomized clinical trials. Int. J. Ophthalmol..

[7] Hou P., Wu D., Nie Y., Wei H., Liu L., Yang G. (2023). Comparison of the efficacy and safety of different doses of atropine for myopic control in children: A meta-analysis. Front. Pharmacol..

[8] Wang J.-D., Liu M.-R., Chen C.-X., Cao K., Zhang Y., Zhu X.-H., Wan X.-H. (2024). Effects of atropine eyedrops at ten different concentrations for myopia control in children: A systematic review on meta-analysis. Eur. J. Ophthalmol..

[9] Lanca C., Repka M.X., Grzybowski A. (2025). Controversies in Myopia Control Treatment: What Does It Mean for Future Research?. Am. J. Ophthalmol..

[10] Wang B., Watt K., Chen Z., Kang P. (2023). Predicting the child who will become myopic—Can we prevent onset?. Clin. Exp. Optom..

[11] Hsieh M.-W., Chang H.-C., Chen Y.-H., Chien K.-H. (2022). Classification-Based Approaches to Myopia Control in a Taiwanese Cohort. Front. Med..

[12] Wnękowicz-Augustyn E., Teper S., Wylęgała E. (2023). Preventing the Progression of Myopia in Children-A Review of the Past Decade. Medicina.

[13] Zhang Y., Su M., Liang L., Shi B., Gong D., Wu Y., Zhang J., Wang M. (2023). The Guiding Significance of Ocular Biometry in Evaluating the Refractive Status of Preschool Children. Ophthalmic Res..

[14] Gaya F., Medina A. (2022). The equations of ametropia: Predicting myopia. J. Optom..

[15] Du L., Ding L., Chen J., Wang J., Yang J., Liu S., Xu X., He X., Huang J., Zhu M. (2025). Efficacy of weekly dose of 1% atropine for myopia control in Chinese children. Br. J. Ophthalmol..

[16] Rose L.V.T., Schulz A.M., Graham S.L. (2021). Use baseline axial length measurements in myopic patients to predict the control of myopia with and without atropine 0.01. PLoS ONE.

[17] Sankaridurg P., Berntsen D.A., Bullimore M.A., Cho P., Flitcroft I., Gawne T.J., Gifford K.L., Jong M., Kang P., Ostrin L.A. (2023). IMI 2023 Digest. Investig. Ophthalmol. Vis. Sci..

[18] Jonas J.B., Ang M., Cho P., Guggenheim J.A., He M.G., Jong M., Logan N.S., Liu M., Morgan I., Ohno-Matsui K. (2021). IMI Prevention of Myopia and Its Progression. Investig. Ophthalmol. Vis. Sci..

[19] Jong M., Jonas J.B., Wolffsohn J.S., Berntsen D.A., Cho P., Clarkson-Townsend D., Flitcroft D.I., Gifford K.L., Haarman A.E.G., Pardue M.T. (2021). IMI 2021 Yearly Digest. Investig. Ophthalmol. Vis. Sci..

[20] Németh J., Tapasztó B., Aclimandos W.A., Kestelyn P., Jonas J.B., De Faber J.H.N., Januleviciene I., Grzybowski A., Nagy Z.Z., Pärssinen O. (2021). Update and guidance on management of myopia. European Society of Ophthalmology in cooperation with International Myopia Institute. Eur. J. Ophthalmol..

[21] Chen J.-W., Chen H.-A., Liu T.-C., Wu T.-E., Lu C.-J. (2024). The Potential of SHAP and Machine Learning for Personalized Explanations of Influencing Factors in Myopic Treatment for Children. Medicina.

[22] Wu T.-E., Chen J.-W., Liu T.-C., Yu C.-H., Jhou M.-J., Lu C.-J. (2024). Identifying and Exploring the Impact Factors for Intraocular Pressure Prediction in Myopic Children with Atropine Control Utilizing Multivariate Adaptive Regression Splines. J. Pers. Med..

[23] Zhang X., Wang Y., Zhou X., Qu X. (2020). Analysis of Factors That May Affect the Effect of Atropine 0.01% on Myopia Control. Front. Pharmacol..

[24] Srivastava O., Tennant M., Grewal P., Rubin U., Seamone M. (2023). Artificial intelligence and machine learning in ophthalmology: A review. Indian J. Ophthalmol..

[25] Oke I., VanderVeen D. (2021). Machine Learning Applications in Pediatric Ophthalmology. Semin. Ophthalmol..

[26] Wy S., Choe S., Lee Y.-J., Bak E., Jang M., Lee S.-C., Ha A., Jeoung J.-W., Park K.-H., Kim Y.-K. (2022). Decision Tree Algorithm-Based Prediction of Vulnerability to Depressive and Anxiety Symptoms in Caregivers of Children With Glaucoma. Am. J. Ophthalmol..

[27] Breiman L., Friedman J.H., Olshen R.A., Stone C.J. (1984). Classification and Regression Trees.

[28] Tibshirani R. (2011). Regression shrinkage and selection via the lasso: A retrospective. J. R. Stat. Soc. Ser. B Stat. Methodol..

[29] Tapasztó B., Flitcroft D.I., Aclimandos W.A., Jonas J.B., De Faber J.H.N., Nagy Z.Z., Kestelyn P.G., Januleviciene I., Grzybowski A., Vidinova C.N. (2024). Myopia management algorithm. Annexe to the article titled Update and Guidance on Management of Myopia. European Society of Ophthalmology in cooperation with International Myopia Institute. Eur. J. Ophthalmol..

[30] Rada J.A., Shelton S., Norton T.T. (2006). The sclera and myopia. Exp. Eye Res..

[31] McBrien N.A., Gentle A. (2003). Role of the sclera in the development and pathological complications of myopia. Prog. Retin. Eye Res..

[32] Sihota R., Tuli D., Dada T., Gupta V., Sachdeva M.M. (2006). Distribution and determinants of intraocular pressure in a normal pediatric population. J. Pediatr. Ophthalmol. Strabismus.

[33] Li Y., Yip M., Ning Y., Chung J., Toh A., Leow C., Liu N., Ting D., Schmetterer L., Saw S.-M. (2024). Topical atropine for childhood myopia control: The Atropine Treatment Long-Term Assessment Study. JAMA Ophthalmol..

[34] Li Y., Wong D., Sreng S., Chung J., Toh A., Yuan H., Eppenberger L.S., Leow C., Ting D., Liu N. (2024). Effect of childhood atropine treatment on adult choroidal thickness using sequential deep learning-enabled segmentation. Asia Pac. J. Ophthalmol..

